# Green-Engineered Montmorillonite Clays for the Adsorption, Detoxification, and Mitigation of Aflatoxin B1 Toxicity

**DOI:** 10.3390/toxins17030131

**Published:** 2025-03-11

**Authors:** Johnson O. Oladele, Xenophon Xenophontos, Gustavo M. Elizondo, Yash Daasari, Meichen Wang, Phanourios Tamamis, Natalie M. Johnson, Timothy D. Phillips

**Affiliations:** 1 Interdisciplinary Faculty of Toxicology, Texas A&M University, College Station, TX 77843, USA; oladelejohn2007@tamu.edu (J.O.O.); gelizondo@exchange.tamu.edu (G.M.E.III); nmjohnson@tamu.edu (N.M.J.); 2 Department of Veterinary Physiology and Pharmacology, College of Veterinary Medicine & Biomedical Sciences, Texas A&M University, College Station, TX 77843, USA; 3 Artie McFerrin Department of Chemical Engineering, College of Engineering, Texas A&M University, College Station, TX 77843, USA; xxenop01@tamu.edu (X.X.); tamamis@tamu.edu (P.T.); 4 Department of Environmental & Occupational Health, Texas A&M University, College Station, TX 77843, USA; 5 Department of Environmental Health Sciences, University of Massachusetts Amherst, Amherst, MA 01003, USA; meichenwang@umass.edu; 6 Department of Materials Science and Engineering, College of Engineering, Texas A&M University, College Station, TX 77840, USA

**Keywords:** aflatoxin, food safety, mycotoxin, enterosorbent, NovaSil, Alphasil, clay, molecular dynamics, isotherms, kinetics

## Abstract

Dietary and environmental exposure to aflatoxins via contaminated food items can pose major health challenges to both humans and animals. Studies have reported the coexistence of aflatoxins and other environmental toxins. This emphasizes the urgent need for efficient and effective mitigation strategies for aflatoxins. Previous reports from our laboratory have demonstrated the potency of the green-engineered clays (GECs) on ochratoxin and other toxic chemicals. Therefore, this study sought to investigate the binding and detoxification potential of chlorophyll (CMCH and SMCH) and chlorophyllin (CMCHin and SMCHin)-amended montmorillonite clays for aflatoxin B1 (AFB1). In addition to analyzing binding metrics including affinity, capacity, free energy, and enthalpy, the sorption mechanisms of AFB1 onto the surfaces of engineered clays were also investigated. Computational and experimental studies were performed to validate the efficacy and safety of the clays. CMCH showed the highest binding capacity (Qmax) of 0.43 mol/kg compared to the parent clays CM (0.34 mol/kg) and SM (0.32 mol/kg). Interestingly, there were no significant changes in the binding capacity of the clays at pH2 and pH6, suggesting that the clays can bind to AFB1 throughout the gastrointestinal track. In silico investigations employing molecular dynamics simulations also demonstrated that CMCH enhanced AFB1 binding as compared to parent clay and predicted hydrophobic interactions as the main mode of interaction between the AFB1 and CMCH. This was corroborated by the kinetic results which indicated that the interaction was best defined by chemosorption with favorable thermodynamics and Gibbs free energy (∆G) being negative. In vitro experiments in Hep G2 cells showed that clay treatment mitigated AFB1-induced cytotoxicity, with the exception of 0.5% (*w*/*v*) SMCH. Finally, the in vivo results validated the protection of all the clays against AFB1-induced toxicities in Hydra vulgaris. This study showed that these clays significantly detoxified AFB1 (86% to 100%) and provided complete protection at levels as low as 0.1%, suggesting that they may be used as AFB1 binders in feed and food.

## 1. Introduction

*Aspergillus fungus*, especially *A. flavus*, generates a class of secondary fungal metabolites called aflatoxins, commonly referred to as mycotoxins. The discovery of aflatoxins emerged after the “Turkey X disease” outbreak in England in 1960, when more than 100,000 turkeys unexpectedly fell ill and perished over several months. The turkeys suffered liver necrosis and extensive intestinal inflammation, according to post-mortem examination. It was quickly revealed that a large number of the turkeys were given Brazilian groundnut meal, which was shown to be highly poisonous when offered to poultry in feeding trials [1]. Later, it was shown that *Aspergillus flavus* metabolites (known as aflatoxins) were the cause of these toxicities. Aflatoxin G2, aflatoxin G1, aflatoxin B2, and Aflatoxin B1 (AFB1) are the four metabolites that are produced by *A. flavus* and are members of the aflatoxin class [2]. According to the International Agency for Research on Cancer (IARC), there is a sufficient indication to categorize aflatoxins as carcinogens in humans, which was discovered after assessing published epidemiological research that showed a substantial correlation between AFB1 ingestion and the development of cancer. The most carcinogenic of the aflatoxins, AFB1, has a long history of being linked to growth retardation, immune system regulation, malnutrition, and hepatocellular carcinoma (HCC) [3].

*A. flavus* is widely distributed in soil and pollutes a variety of crops around the globe, including peanuts, rice, and corn. Controlling aflatoxin-related contamination is challenging because fungus can grow on food at any time before or after harvest. Furthermore, nations with high humidity and temperatures, such as Southeast Asia and Sub-Saharan Africa, tend to have higher levels of AFB contamination since these regions encourage the growth of fungi. Furthermore, a significant amount of AFB1 exposure happens during storage because many of these nations lack the capacity to preserve food in dry, temperature-controlled settings [4,5,6]. About 4.5 billion people in underdeveloped nations were considered to be at risk for chronic, unregulated exposure to aflatoxins in 2004 [7,8], underscoring the challenges of preventing aflatoxin contamination. The agricultural sector suffers significant losses every year as a result. Numerous researchers have calculated the annual losses resulting from crop contamination with aflatoxin in different nations. Between USD 20 million and USD 2 billion have been estimated to be lost annually in the United States [9,10,11]. Both Asian and African nations suffer significant losses. Thailand, Indonesia, and the Philippines were predicted to lose USD 1 billion a year if the European guidelines of aflatoxin contamination were imposed, whereas African exporters were predicted to lose USD 670 million, or 64% of their revenues [12,13]. Additionally, it has been predicted that nations may begin to see larger levels of AFB1 contamination in crops, and that new locations may begin to experience issues with AFB1 exposure as climate change influences average temperatures and annual rainfall [14,15]. Therefore, remedies to AFB1 exposure and toxicities must be developed before aflatoxins become an even more serious worldwide health and economic issue.

Research activities in the laboratory of Dr. T.D. Phillips over the past four decades have pioneered and explored various mitigation and detoxification approaches using materials that are generally recognized as safe (GRAS) to reduce the exposure and toxicities of aflatoxins, both in animals and humans [16,17,18,19,20,21]. These studies include the development of an aflatoxin enterosorbent to inhibit the adsorption of AFB1 in the gastrointestinal tract, reducing its uptake into the circulatory system and preventing its delivery to target organs, which is responsible for its major toxicity. This work has led to commercialization of aflatoxin binders worldwide, including NovaSil (a calcium montmorillonite clay) and Alphasil (a sodium montmorillonite clay). The addition of these clays to animal feeds has been shown to reduce AFB1 absorption and AFM1 levels in milk, establishing proof-of-concept and the protective ability of these clays against AFB1 toxicities in a number of experimental animals. In many animal models, NovaSil clay significantly reduced the harmful effects of aflatoxin-contaminated diets in farm animals [16,17]. A long-term trial in rodents was also conducted, in which rats were given up to 2.0% *w*/*w* of NovaSil for 28 weeks. According to Afriyie-Gyawu et al. [18], there were no detectable toxic effects of the clay observed throughout this investigation, suggesting that this clay would be safe to use in further work.

Following a Phase I dose titration study in human participants in Texas, selected oral doses of a placebo and low and high doses of refined NovaSil clay were administered by capsule to Ghanaians living in an area at high risk for AFB1 exposure. After three months, participants in the low- and high-dose groups showed significantly decreased serum AFB1-albumin and urine AFM1 biomarkers. Furthermore, only minor adverse effects (such as nausea, diarrhea, heartburn, and dizziness) were recorded in both placebo and treatment groups, and NovaSil was not demonstrated to significantly change the health parameters of study participants [19,20]. These findings suggested that incorporating NovaSil into the diet may be effective way to diminish AFB1 toxicity. Further clinical trials in Africa showed that NovaSil was safe and effective when administered in the diet and in flavored water [21].

There is a high possibility that aflatoxins coexist with other mycotoxins and environmental chemicals such as benzene, pesticides, and PFAS. Thus, there is a critical need for the development of sorbent materials that will bind both mycotoxins and environmental chemicals. We have recently shown that the addition of various forms of chlorophyll to NovaSil clay can result in clays with enhanced hydrophobicity and attraction for lipophilic mycotoxins like ochratoxin A and zearalenone and chemicals such as PFAS and benzene [22,23,24,25].

In this current study, we investigated the activity of green-engineered clays (GECs) to bind and mitigate mixtures of aflatoxin B1. More specifically, aflatoxin B1 binding and interactions were assessed using in vitro, in silico, in vivo, and cell line models to achieve the following: (i) evaluate the adsorption characteristics of AFB1 on the surfaces of GECs using well-established methods of isothermal analyses, (ii) assess the kinetic and thermodynamic profile of AFB1 binding to and desorption from the clays, (iii) explore the atomistic dynamics and binding modes of the binding using computational approaches based on molecular dynamics simulations, (iv) determine the protective efficiency of GECs in hepatic cells, and (v) validate the safety and efficacy of GECs in living organisms (*Hydra vulgaris*).

## 2. Results

### 2.1. Isothermal Adsorption of AFB1 on the Clays at pH2 (Gastric Condition)

**Figure 1:**
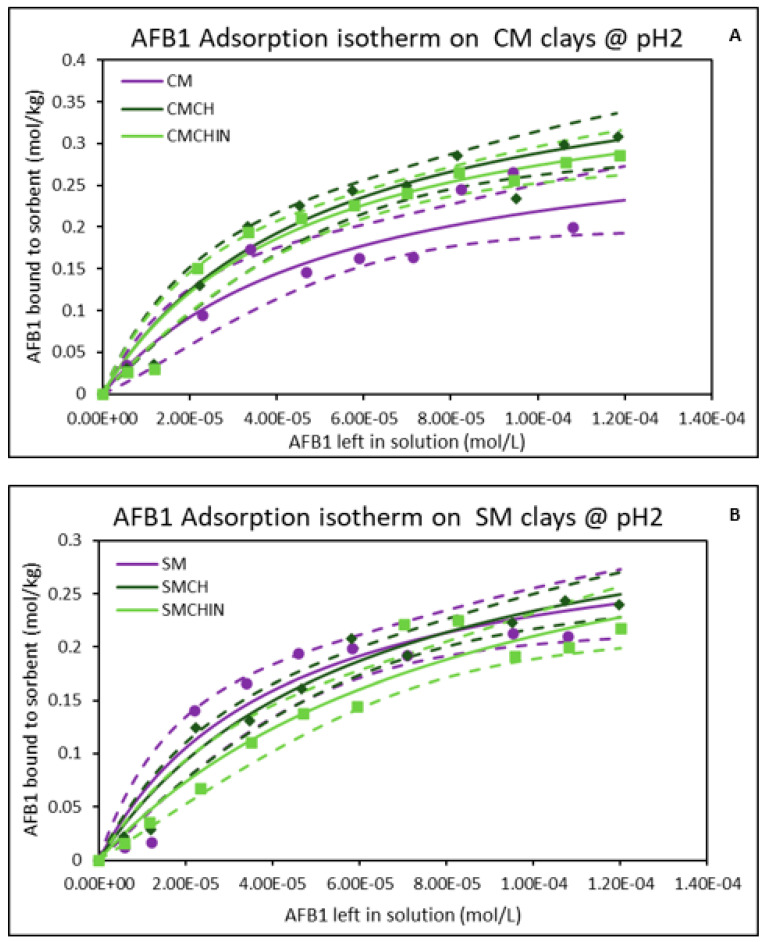
Adsorption isotherms of AFB1 onto binding surfaces of (**A**) CM-clays and (**B**) SM-clays at pH2. SMCHin; chlorophyllin-amended sodium montmorillonite; SMCH: chlorophyll-amended sodium montmorillonite; SM: sodium montmorillonite; CMCHin; chlorophyllin-amended calcium montmorillonite; CMCH: chlorophyll-amended calcium montmorillonite; CM: calcium montmorillonite.

The results shown in Figure 1 demonstrate the isothermal adsorption of AFB1 on both the parent montmorillonites and the GECs at pH2 and 37 °C. The isothermal plot of AFB1 on both parent montmorillonites (CM and SM) fit the Langmuir model, and the curved shapes indicate that AFB1 binding was saturable and tight on these clay surfaces. The binding capacities (Qmax) of CM and SM for AFB1 were 0.34 mol/kg and 0.32 mol/kg, respectively, while their binding affinities (Kd) were 1.89 × 10^4^ and 2.40 × 10^4^, respectively. The adsorption isotherm of AFB1 for GECs showed that the amendment of montmorillonites with chlorophyll and chlorophyllin improved their binding capacities. Chlorophyll increased the Qmax of CM and SM to 0.43 mol/kg and 0.38 mol/kg, respectively. Similarly, chlorophyllin enhanced the Qmax of CM (0.40 mol/kg) and SM (0.39 mol/kg). The Gibbs free energies (∆G) of the adsorption of AFB1 onto the clay surfaces were between −19.19 kJ/mol to −20.28 kJ/mol, indicating chemosorption. A summary of all the adsorption parameters is displayed in Table 1.

### 2.2. Isothermal Adsorption of AFB1 on the Clays at pH6 (Intestinal Condition)

**Figure 2:**
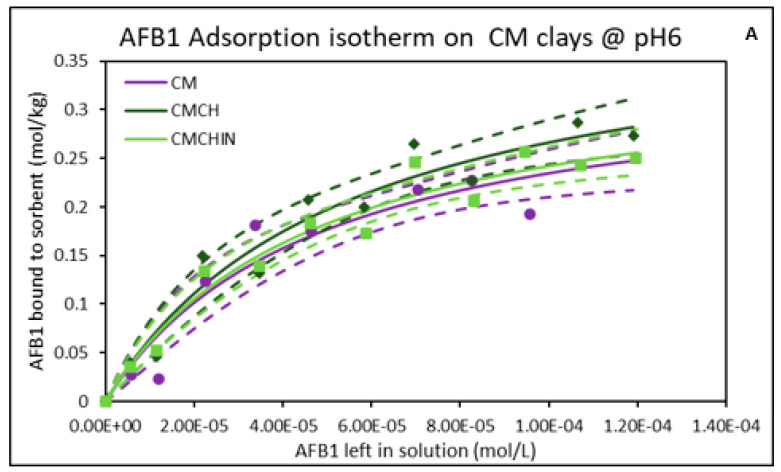

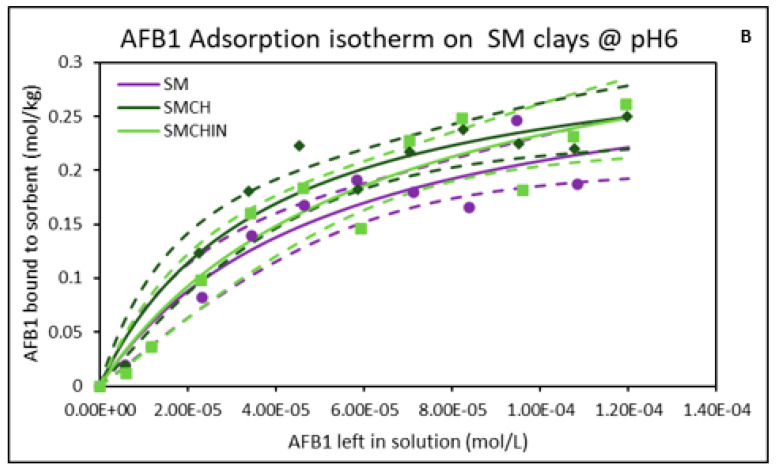
Adsorption isotherms of AFB1 onto binding surfaces of (**A**) CM-clays and (**B**) SM-clays at pH6. SMCHin; chlorophyllin-amended sodium montmorillonite; SMCH: chlorophyll-amended sodium montmorillonite; SM: sodium montmorillonite; CMCHin; chlorophyllin-amended calcium montmorillonite; CMCH: chlorophyll-amended calcium montmorillonite; CM: calcium montmorillonite.

At pH6 (intestinal pH) and 37 °C, the adsorption plots of AFB1 binding on the surfaces of the parent montmorillonites (CM and SM) exhibited good fit to a Langmuir model based on correlation coefficients and a curved shape, suggesting the presence of monolayer binding and saturable active sites (Figure 2). The binding of AFB1 onto parent montmorillonites and green-engineered clays showed high binding capacities of 0.35 and 0.31 mol/kg for CM and SM, respectively. Higher binding capacities ranging from 0.33 to 0.41 mol/kg were recorded for the green-engineered clays with chlorophyll-amended clay (CMCH) having the highest capacity of 0.41 mol/kg. Furthermore, all the clays exhibited high affinities in the magnitudes of 10^4^ (Table 1). Interestingly, these results showed that the change in pH did not affect the binding of AFB1 on the surfaces of the montmorillonite clays. The binding potency of these clays was enhanced following amendment with chlorophyll and chlorophyllin, with increased Qmax, as shown in Table 1. Remarkably, the chlorophyll-engineered clays exhibited higher binding capacities than the chlorophyllin clays.

### 2.3. Kinetics of the Binding of AFB1 on the Clays

**Figure 3:**
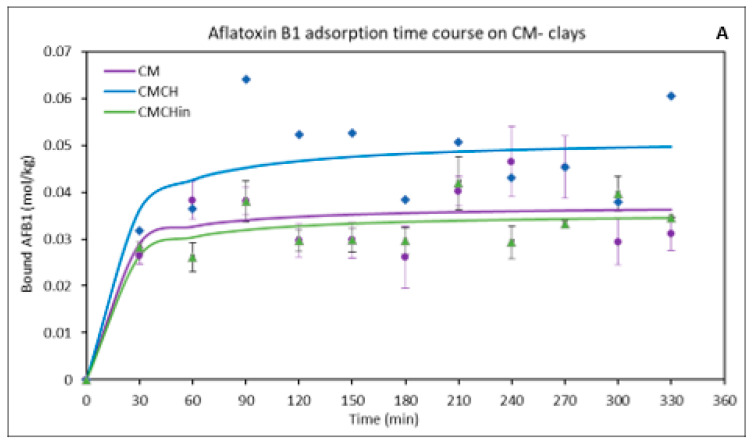

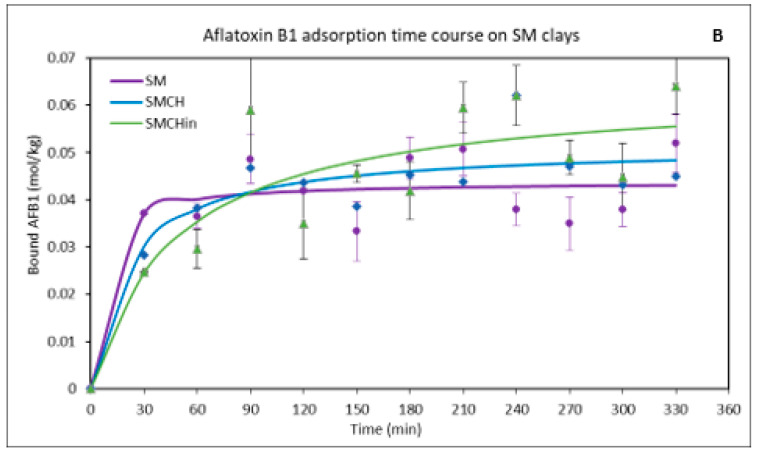
Effect of contact time on the adsorption of AFB1 on binding surfaces of (**A**) CM-clays and (**B**) SM-clays. SMCHin; chlorophyllin-amended sodium montmorillonite; SMCH: chlorophyll-amended sodium montmorillonite; SM: sodium montmorillonite; CMCHin; chlorophyllin-amended calcium montmorillonite; CMCH: chlorophyll-amended calcium montmorillonite; CM: calcium montmorillonite.

Nonlinear pseudo-first-order, pseudo-second-order, and Elovich models were used to better understand the AFB1 adsorption kinetics on the binding surfaces of the parent and engineered clays. The adsorption of AFB1 onto the CM clays (Figure 3A) and SM clays (Figure 3B) sorbents’ binding surfaces was best described by the pseudo-second-order kinetic model, according to the correlation coefficient value and the consistency between the modeled values (qe, cal) and experimental binding capacity (qe, exp). Table 2 displays the kinetic model’s parameters. The greatest binding capacity (qe, exp) values are 0.08 mg/kg for CMCH, 0.07 mg/kg for SMCH and SMCHin, and 0.05 mg/kg for CM and SM.

### 2.4. In Silico Molecular Dynamics (MD) Simulations Investigating AFB1 Binding to Surfaces of Clay (CM) and Chlorophyll-Engineered Clay (CMCH)

**Figure 4:**
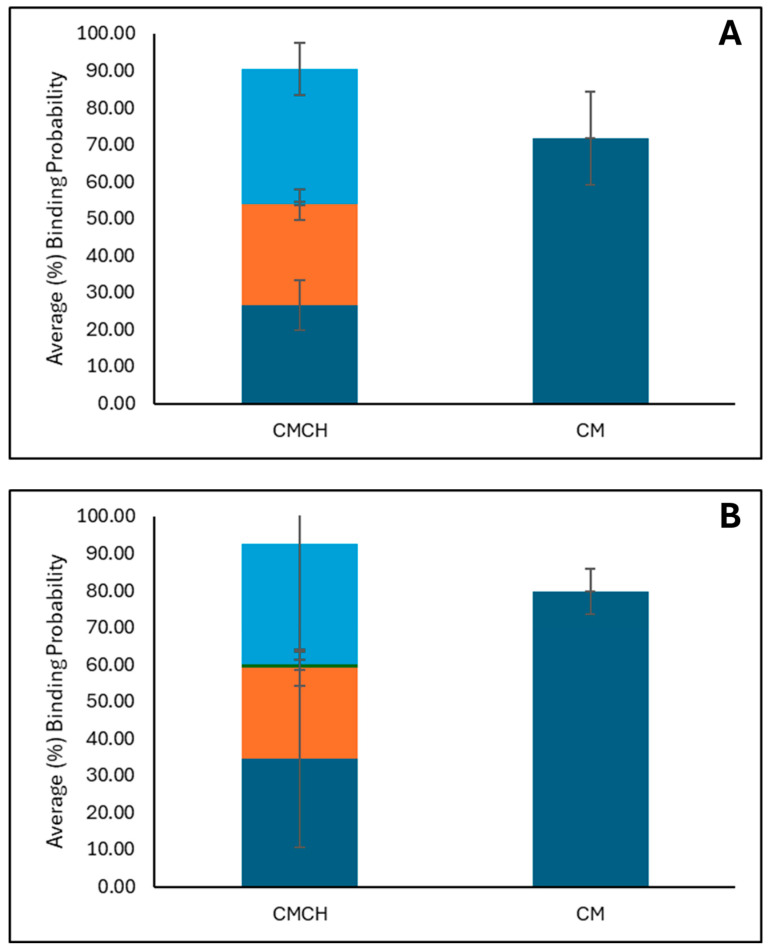
The panels (**A**,**B**) show the average percentage binding probability of AFB1 at pH2 and pH7, respectively, to chlorophyll-amended clay (CMCH) and parent clay (CM). Direct, direct-assisted, direct-helped, and indirect-assisted interactions are shown in dark blue, orange, green, and cyan, respectively. The average values are calculated from triplicate runs. The error bars denote the standard deviation values calculated from triplicate runs.

Figure 4 shows the average binding percentage probability of AFB1 to CMCH and CM at both pH2 and pH7 (Figure 4A,B, respectively). Both CM and CMCH showed high binding probability >70% at both pH conditions. CMCH showed a ~20% increase in the binding probability when compared to the parent clay at pH2 and a ~10% increase at pH7. The total binding capacity of AFB1 to CM and CMCH at both pH conditions is highly similar to the slightly higher probability occurring at pH7 compared to pH2 for CM and direct interactions in CMCH. This could be attributed to the slightly higher tendency of AFB1 to participate in contacts with the edges in the clay surfaces of the neutral clay compared to the acidic clay surfaces. CMCH variably contributed to AFB1’s binding due to both direct-assisted and indirect-assisted interactions (Figure 4).

**Figure 5:**
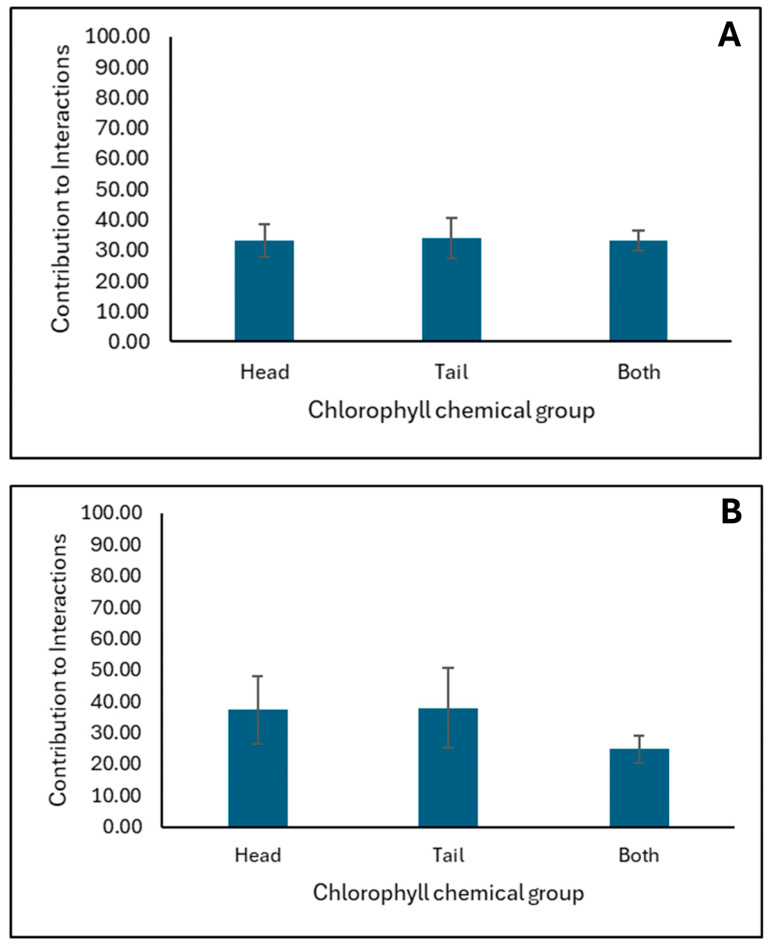
The panels (**A**,**B**) show the average contribution of chlorophyll’s chemical groups to the interactions with AFB1 at pH2 and pH7, respectively. The average values are calculated from triplicate runs. The error bars denote standard deviation values calculated from triplicate runs.

Figure 5 presents the percentage contribution of each chlorophyll chemical group to the interactions with AFB1 at both pH2 and pH7 (Figure 5A,B, respectively). The interaction between AFB1 and chlorophyll’s chlorin ring (head) or alkyl chai (tail) is approximately equally probable at both pH conditions within the simulations.

### 2.5. Cytotoxicity Effect of AFB1 on Hep G2 and Protective Ability of Green-Engineered Clays

**Figure 6:**
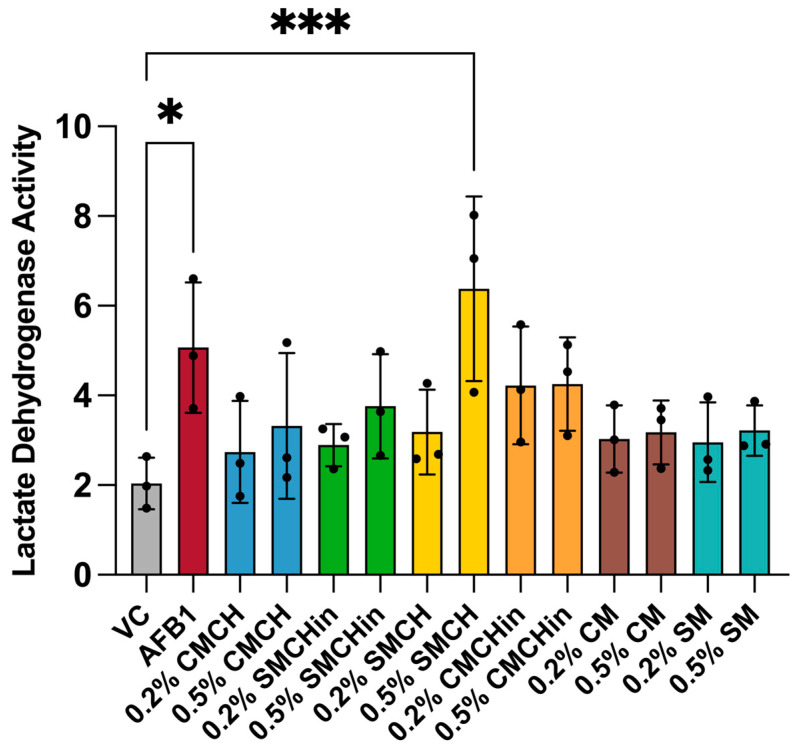
Cytotoxicity of aflatoxin B1 and effects of clay treatments in Hep G2 cells measured by LDH release after 24 h exposure. Relative amount of LDH released into cell culture medium was determined after 24 h exposure to either 20 ppm aflatoxin B1 or clay-treated media containing aflatoxin-1 B and compared to vehicle control. Bars are color-coded to represent vehicle control, aflatoxin B1, and six different clays, with each clay having two dose levels. Individual data points are shown as circles. Values are presented as mean ± standard deviation (SD). N = 3 for each treatment. Significant differences are indicated by (*) for *p* < 0.05 and (***) for *p* < 0.001.

AFB1 exposure caused significantly elevated levels of LDH in Hep G2 cells, reflecting hepatic toxicity (Figure 6). Each clay-treated exposure condition did not show significantly elevated levels of LDH in spent media, with the exception of 0.5% (*w*/*v*) SMCH. Generally, there appeared to be a protective effect associated with clay treatment.

### 2.6. Effect of AFB1 on Hydra Vulgaris and Detoxification Efficacy of Green-Engineered Clays

**Figure 7:**
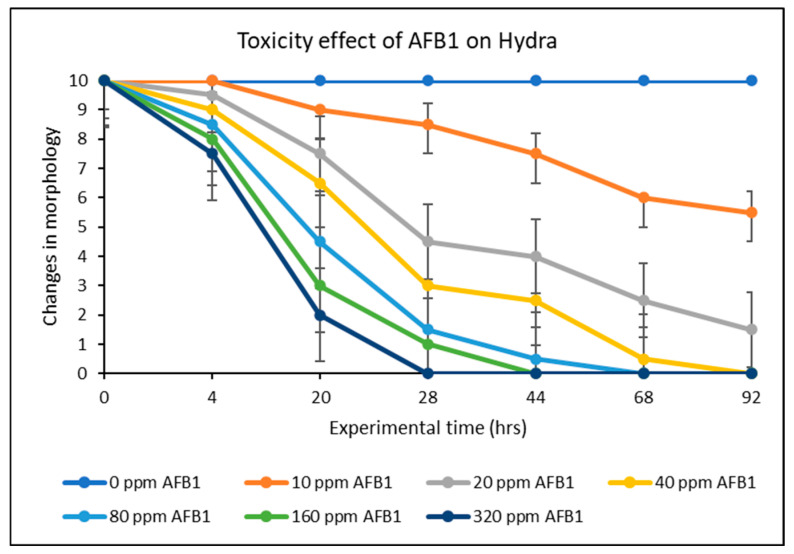
Toxicity effects of AFB1 exposure to hydra.

**Figure 8:**
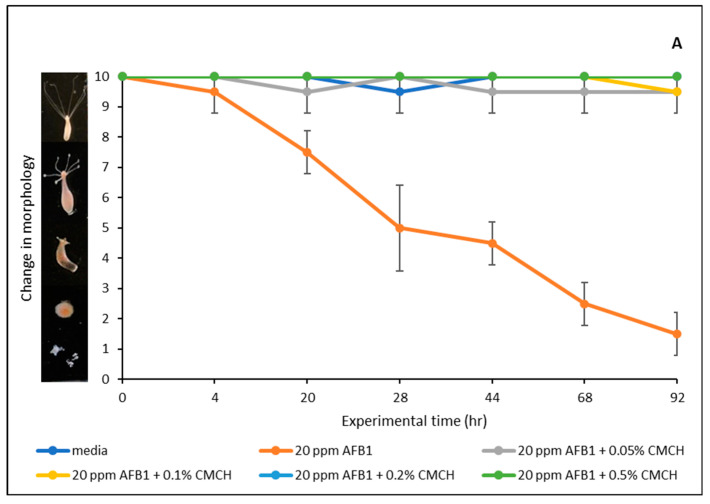

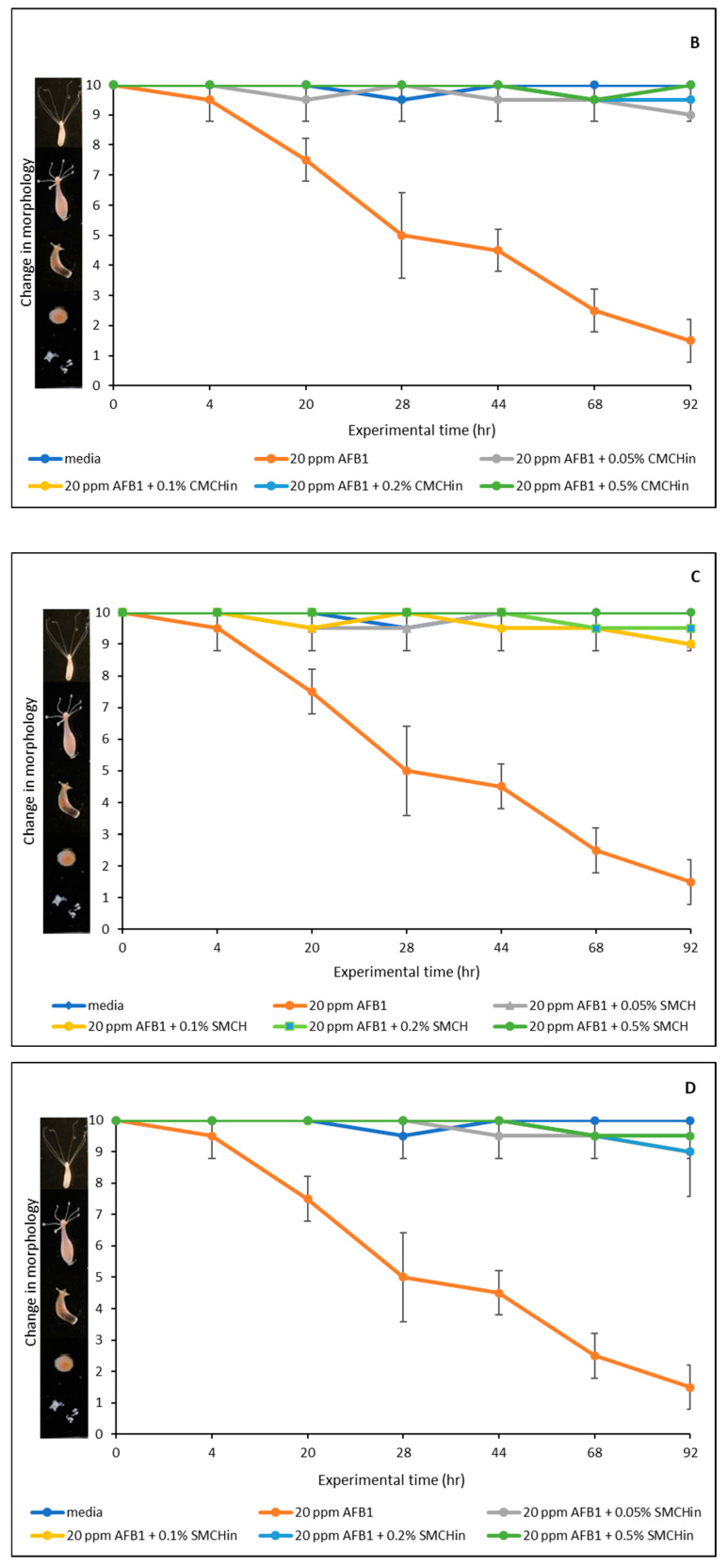

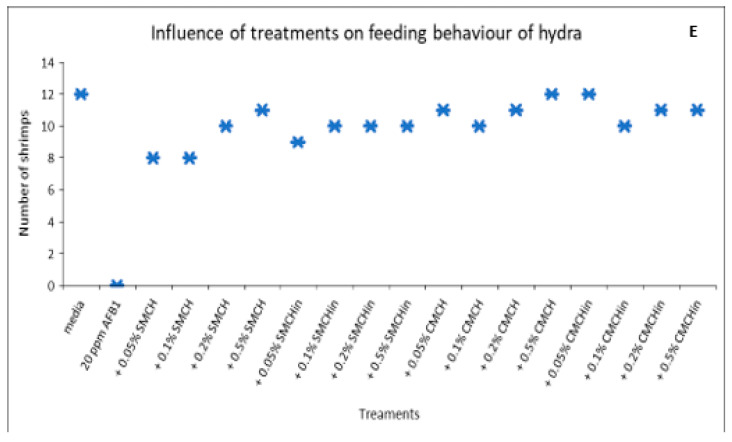
Protective effects of green-engineered clays on AFB1-toxicities to hydra (**A**) with chlorophyll-amended calcium montmorillonite; (**B**) with chlorophyllin-amended calcium montmorillonite; (**C**) chlorophyll-amended sodium montmorillonite; (**D**) chlorophyllin-amended sodium montmorillonite. (**E**) Influence of treatment on feeding behavior of hydra.

Figure 7 displays the findings of the AFB1 toxicity assessment conducted on hydra in vivo. AFB1 exposure between 10 and 200 ppm showed significant toxicity to hydra morphology. In particular, AFB1 impaired the hydra’s morphology between 50 and 100 percent at different doses of 10, 20, 40, 80, 100, and 200 ppm. Notably, after 48 h of exposure, the hydra subjected to concentrations greater than 20 ppm of AFB1 completely disintegrated. Therefore, 20 ppm was used for the detoxification studies and evaluation. The hydra colony was considerably shielded against AFB1 toxicity by treatment with different doses of the montmorillonite-clay-based sorbent additives, ranging from 0.05% to 0.5% (*w*/*v*), as seen in Figure 8. In particular, protection ranged from 33% to 93% for SM-amended clays (SMCH and SMCHin) at 0.2% and 0.5% inclusion, whereas it ranged from 87% to 100% for CM-amended clays (CMCH and CMCHin). Interestingly, CMCH provided full (100%) protection at both 0.2% and 0.5% inclusion. The feeding behavior of Hydra vulgaris was significantly impaired by 20 ppm of AFB1; however, the inclusion of the clays prevented this toxicity.

## 3. Discussion

The inclusion of selected clays to diet are novel ways to address AFB1 contamination [26,27,28,29]; they work as binding agents to reduce the bioavailability of AFB1 from the gastrointestinal tract following consumption, hence preventing AFB1’s uptake by the blood and effects in the body. As shown in the results of this study, both the parent clay and GECs significantly reduced the amount of aflatoxin in the simulated gastrointestinal fluids. Previous studies in our laboratory have established the parent clay (NovaSil) as an excellent binder of aflatoxin [20,21,30,31,32,33]. Interestingly, the amendment of these clays with both chlorophyll and chlorophyllin increased the binding capacities of the clays. Interestingly, the inclusion of chlorophyll and chlorophyllin as dietary supplements in human foods and animal diets has been reported to decrease systemic absorption of aflatoxins. According to Breinholt et al. [34], the AFB1-DNA adduct in rainbow trout was reduced by 37% when chlorophyllin was added to contaminated feed, resulting in a 77% decrease in the prevalence of cancer [34]. Similarly, the addition of chlorophyllin to AFB1-contaminated diet reduced AFM1 in rat urine by 90%, AFB1-DNA adducts by 42%, and AFB1-albumin adducts by 65% [35]. Furthermore, chlorophyll also demonstrated efficacy in the same trial by lowering urinary AFM1 levels by 92%, AFB1-DNA adducts by 55%, and AFB1-albumin adducts by 51% [35]. A human study also observed that adding chlorophyllin to the diets of those living in high-risk locations for AFB1 exposure decreased AFB1-N7-guanine levels by 55% when compared to those who did not receive chlorophyllin [36].

In this study, chlorophyll-amended clays showed a higher binding capacity for aflatoxin B1 than the parent clays. Our observation is similar to animal and clinical studies that used chlorophyll and chlorophyllin compounds as preclinical interventions. When chlorophyllin and chlorophyll were added to aflatoxin B1-contaminated diets, the tumor incidence in the experimental rats decreased by 74 and 77%, respectively [35]. Also, four participants in a clinical study involving human volunteers received a single dose of 30 ng of AFB1 three times. Chlorophyll and chlorophyllin were given together with the AFB1 dose for the second and third doses. According to the findings, chlorophyll decreased urinary AFM1 levels by 41% and chlorophyllin by 28% [37].

**Figure 9:**
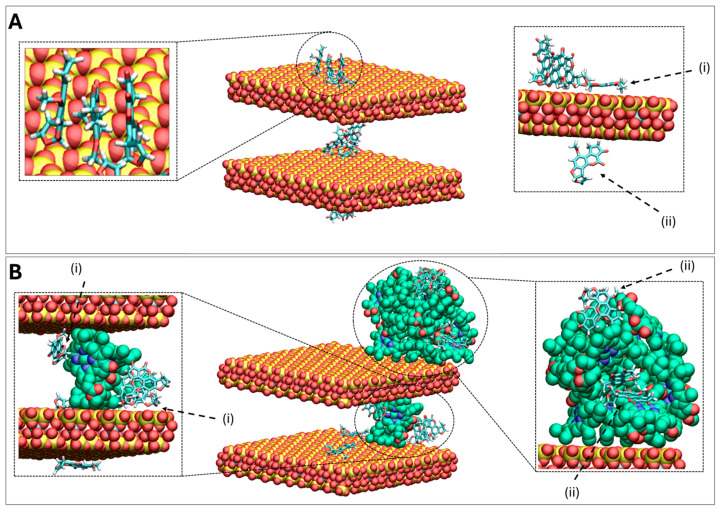
Panels (**A**,**B**) show the last snapshot of a particular simulation of AFB1 in complex with CM and CMCH, respectively, at pH2. The pictures in the center of the panels are zoomed-out representations while the picture on the left and the right of the panels correspond to zoomed-in representations, with different angles shown for clarity. The clay layers are shown in vdW representation, colored by atom type. The carbon atoms of chlorophyll amendments are colored in green, while the magnesium, nitrogen, and oxygen atoms are colored by atom type. The AFB1 molecules are shown in licorice representation, colored by atom type. The calcium ions that are within a distance of 3.5 Å of clay, chlorophyll, or AFB1 are shown in vdW representation, colored in tan. The hydrogen atoms of clay and chlorophyll molecules were omitted for clarity. The indicated molecules (i) and (ii) are discussed in the main text.

Previous research has demonstrated that parent clays (calcium montmorillonite, CM and sodium montmorillonite, SM) are highly effective binders for AFB1 and significantly mitigate toxicity when included in aflatoxin-contaminated animal feed. It is noteworthy that these base clays are now included as aflatoxin binders in feed worldwide. Importantly, the findings from this study for GECs revealed better binding than the parent montmorillonite clays. Isothermal analyses indicated that the binding of AFB1 to these clays was high affinity and high capacity. Computational studies demonstrated that AFB1 could interact with both the ring and long alkyl tail of the chlorophyll-engineered clay, suggesting the key role of hydrophobic interactions between the two moieties. The final simulation snapshot from one of triplicate runs performed for AFB1 in complex with CM and CMCH at acidic conditions are shown in Figure 9 (Panels A and B, respectively). In CM, AFB1 depicted an overall increased tendency to aggregate by stacking in parallel and form a cluster interacting with the clays, shown in Figure 9A with a dashed circle. Individual AFB1 molecules also interacted with clay by either lying flat on its surface or with the methyl groups, shown in Figure 9A with dashed arrows (i) and (ii), respectively. In CMCH, AFB1 clusters additionally participated in primarily hydrophobic interactions with chlorophyll amendments of CMCH, both via direct-assisted and indirect-assisted interactions, as shown in Figure 9B with dashed arrows (i) and (ii), respectively.

Furthermore, the binding ability of these GECs has been established with hazardous lipophilic environmental substances such as benzene, toluene, and xylene, and lipophilic mycotoxins such as ochratoxin A [23,24,25]. This highlights the added benefits of these clays in real-world scenarios containing mixtures of these and other toxic chemicals. AFB1 treatment significantly elevated the levels of LDH in spent media after 24 h exposure; however, AFB1-induced cytotoxicity was mitigated in hepatic cells after treatment with GECs, with the exception to this being clay 0.5% (*w*/*v*) SMCH. The next steps would be to repeat the exposures and assess a time point up to 72 h with media collection each 24 h to assess LDH activity in spent media. Also, the inclusion of clay treatments alone to assess the cytotoxicity of the clay types on liver cells would also be beneficial.

The safety and protective capacities of GECs were determined utilizing the hydra assay. As shown in the studies, all the clays at inclusion rates ranging from 0.05 to 0.5% (*w*/*v*) provided significant protection to hydra against AFB1 toxicity. The in vivo protection supports the in vitro isothermal findings. The >99% protection offered by these clays suggests that clay amendments with chlorophyll and chlorophyllin are safe and provide excellent protection against AFB1 in adult hydra.

## 4. Conclusions

This study evaluated the capacity of GECs to adsorb and detoxify aflatoxin B1. In vitro, in silico, and in vivo techniques were used to evaluate the binding properties of these green organoclays. At the physiological pH of the gastrointestinal tract, in vitro isothermal analyses revealed that GECs had significantly higher adsorption capacities, affinities, and free energy levels of sorption for aflatoxin B1 versus the parent clays. The results suggested that chemosorption was the mechanism by which aflatoxin B1 was bound to the GECs. The sorption and detoxification of aflatoxin B1 was confirmed by the notable protection against aflatoxin B1 toxicity. The combined in vitro and in silico findings provided a distinct perspective on the aflatoxin B1-binding processes on the surfaces of the clays. The in vivo investigations confirmed the effectiveness and efficacy of the clays. Taken together, the findings of this study suggested that these clays may be used for the mitigation of aflatoxin B1 toxicities during acute outbreaks of aflatoxicosis and may reduce unintentional exposures to people and animals from contaminated food and feed. Moreover, these chlorophyll-amended clays have been shown to sequester and neutralize environmental chemicals such as benzene, toluene, ochratoxin A, and other hazardous substances, which will enhance their impact on health.

## 5. Materials and Methods

### 5.1. Reagents and Chemicals

AFB1 was acquired from Sigma Aldrich, located in Saint Louis, MO, USA. Aldrich Chemical Co. (Milwaukee, WI, USA) supplied the sulfuric acid (H_2_SO_4_, 95–98%) and chlorophyllin. The source of the chlorophyll was Santa Cruz Biotechnology, located in Dallas, TX, USA. Acetonitrile, for high-pressure liquid chromatography (HPLC) quality and pH buffers (4.0, 7.0, and 10.0), was obtained from VWR (Atlanta, GA, USA). Human hepatocellular carcinoma (Hep G2) cells were obtained from ATCC, (Manassas, VA, USA) with product number HB-8065. The Elga^TM^ automatic filtration system (Woodridge, IL, USA) was utilized to produce ultrapure deionized water (18.2 MΩ), which was used in the experiments. The Source Clay Minerals Repository at the University of Missouri–Columbia provided sodium montmorillonite (SM), which has a cation exchange capacity of 75 cmol kg^−1^. Engelhard Corp. (Cleveland, OH, USA) provided the calcium montmorillonite (CM), which has a cation exchange capacity of 97 cmol kg^−1^, an exterior surface area of about 70 m^2^ g^−1^, and an average total surface area of up to 850 m^2^ g^−1^ [38]. Both clays are represented by the general formula (Na,Ca)_0.3_(Al,Mg)_2_Si_4_O_10_(OH)_2_·nH_2_O. In addition to mesopores of around 5 nm in diameter, the samples also contained some mica, quartz, sanidine, and calcite as impurities [30].

### 5.2. Synthesis and Amendment of GECs

Previously established methods from our laboratory [22,24] were used to amend the parent clays (CM and SM). Briefly, at 150% cation exchange capacity, the SM and CM clays were modified with water-soluble chlorophyllin to yield hydrophilic SMCHin and CMCHin, respectively, and with water-insoluble chlorophyll to develop organoclays, SMCH and CMCH, respectively. To help oversaturate exchangeable sites with chlorophyllin or chlorophyll, SM or CM at 5% *w*/*w* in pH 4.2 water were swiftly agitated with chlorophyll or chlorophyllin for 24 h. Following a 20 min centrifugation at 3000× *g*, the suspensions were rinsed with distilled water. Following three iterations of the washing procedure, each sample was dried in a desiccator before being ground up and run through a 150 µm sieve. The stability of chlorophyll in clay interlayers and the full characterization of these clays including light sensitivity, lipophilicity, expansibility in water, particle size, zeta potential, scanning electron microscopy (JSM-7500F, JEOL, Peabody, MA, USA), X-ray powder diffraction (Bruker D8 Endeavour, Bruker, Karlsruke, Germany), and Fourier-transform infrared spectroscopy (IRPrestige-21, Shimadzu, Japan) has been previously established in our laboratories [22,25]. The physicochemical characteristics such as pH, moisture content, coefficient of expansibility in water, hydrophobicity, bulk density, and pHpzc have also been reported [24].

### 5.3. Sorption Isotherms at Physiological pHs (pH2 and 6)

To replicate gastric acid and intestinal neutral pH conditions, adsorption isotherms were performed at pH2 and 6 following the procedure of Oladele et al. [23]. Briefly, pure crystals of AFB1 were dissolved in acetonitrile to make its stock solution. A UV-visible spectrophotometer was used to scan and read the absorbance at 362 nm after a calculated amount of the stock solution was injected into distilled water with a pH of 2 or 6 to prepare an 8 ppm AFB1 solution. In order to prevent AFB1 precipitation in the solutions, the maximum AFB1 concentration was fixed at 8 ppm, which is significantly below the solubility range of 11–33 ppm. Next, a gradient of increasing concentrations of AFB1 solution (ranging from 0.4 ppm to 8 ppm) was applied to 100 μg of sorbents. To prepare the concentration gradient of AFB1 solutions, polypropylene centrifuge tubes were filled with a determined volume of 8 ppm solution. A corresponding volume of distilled water (5 mL) followed by 50 μL of a 2 mg/mL clay solution were injected to accomplish the 100 μg sorbent inclusion. The suspension was thoroughly mixed using an autopipette throughout the transfer to the sorbent/toxin mixture. Three tubes containing 8 ppm AFB1 solution, distilled water, and 100 μg sorbent in distilled water served as the three controls. An electric shaker was used to shake the test and control groups for two hours at 37 °C and 1000 rpm. After that, all samples were centrifuged for 20 min at 2000× *g* in order to separate the clay/AFB1 complex from the solution. Samples and controls were tested for AFB1 absorption in the supernatant using a UV-visible spectrophotometer.

### 5.4. Adsorption Kinetics

The ability of green clays to bind AFB1 over a 6 h exposure time was assessed in this study using adsorption kinetic models. A 100 ug aliquot of each GEC was combined with 8 ppm of AFB1 in pH2 water at 37 °C. After agitating the mixture at 800 rpm, the residual AFB1 concentration was measured using a UV-visible spectrophotometer at 30 min intervals to examine the adsorption kinetics. To analyze the adsorption dynamics, the study looked at pseudo-second-order, pseudo-first-order, and Elovich models [39,40].

### 5.5. Data Calculations and Curve Fitting

Using Beer’s law, the concentration of AFB1 remaining in solution (c) was determined using the UV-visible absorption data. The concentration difference between the test and control groups was used to compute the quantity adsorbed for each data point. More precisely, the quantity of AFB1 bound by sorbents (in mol/kg) was represented on the y-axis. It was computed by dividing the mass of the contained clays by the difference between the moles of free AFB1 in the test solution and control groups.

Beer’s law: absorbance = *eLC* (1)

where e is the molar extinction coefficient (e for AFB1 = 21,865 cm^−1^ mol^−1^), and L is the path length of the cell holder = 1 cm, dependent on the cuvette.

Table-curve 2D V. 5.01 (Systat Software, Inc., Palo Alto, CA, USA) and the R programming language were employed to examine the adsorption data and ascertain specific parameters. The R code was used to calculate the adsorption data, and maximum likelihood estimation was used to confirm that the data conformed to well-known and accepted models. Confidence intervals and standard errors were calculated using the information matrix approach [41,42].

Adsorption isotherms based on the mean of the observed data points and the 95% confidence intervals from triplicate studies were displayed using the Langmuir model. The Langmuir model illustrates the adsorption of a monolayer on a surface with uniform adsorption energies and a finite number of identical sites. In an ideal Langmuir scenario, adsorption sites with the same energy level suggest a homogenous surface with the least amount of interaction between the adsorbed species.

(2) $$\mathrm{Langmuir}\;\mathrm{model}\;q=Q_{\max}\;\frac{K_{d}C_{w}}{1+K_{d}C_{w}}$$

where *C*_w_ = equilibrium concentration of AFB1 (mol L^−1^), *K*_d_ = Langmuir distribution constant, *Q*_max_ = maximum binding capacity (mol kg^−1^), and *q* = the amount of AFB1 adsorbed (mol kg^−1^).

To determine *Ke°* from *K_d_*, the following equation was used:

(3) $$\mathrm{Ke}{}^{\circ}=\frac{K_{d}{\left[adsorbate\right]}^{o}}{\gamma }$$

where *Ke°* is the thermodynamic equilibrium constant, [*adsorbate*]° is the standard concentration of the adsorbate = 1 mol L^−1^, and *γ* is the coefficient of activity.

The enthalpy (Δ*H*) and free energy (Δ*G°*) were determined by using the Gibbs free energy equation together with the adsorption parameters and van’t Hoff equation as given below:

ΔG = ΔG° + RTInKe°(4)

(5) $$\Delta H=\frac{-RIn(\frac{K_{d2}}{K_{d1}})}{\left(\frac{1}{T_{2}})-(\frac{1}{T_{1}}\right)}$$

where *T* (absolute temperature) = 273 + *t* (°C) and *R* (gas constant) = 8.314 J mol^−1^ K^−1^. When Δ*G°* is positive, the adsorption process is not favored and cannot be significant (Ke° < 1). However, if the value is negative, it is an indication that the adsorption process is enhanced thermodynamically and is proceeding forward (Ke° > 1). Δ*G* will be zero for an adsorption system in equilibrium.

The expression for the nonlinear pseudo-first-order rate equation is displayed as follows:

q_t_ = q_e_ × [1 − exp (−K_1_ × t)] (6)

where *q_e_* and *q_t_* denote the amounts in mg/kg of AFB1 adsorbed onto the binding surfaces of various sorbents at equilibrium and time *t* (min). The rate constant of the first order is *K*_1_ (min^−1^).

The expression for the nonlinear pseudo-second-order rate equation is as follows:

(7) $$q_{t}=\frac{K_{2}\times {q_{e}}^{2}\times t}{1+q_{e}\times K_{2}\times t}$$

where *q_e_* and *q_t_* denote the amounts in mg/kg of AFB1 adsorbed onto the binding surfaces of various sorbents at equilibrium and time *t* (min). The rate constant of the second order is *K*_2_ (mg kg^−1^ min^−1^).

The expression for the Elovich equation is as follows:

q_t_ = 1/b ln ab + 1/b ln t (8)

This study involves calculating the initial adsorption rate represented by the symbol a (in mg/kg min) and the parameter b, which are related to the amount of surface covered and the activation energy involved in chemisorption (in mg kg^−1^). The kinetic parameters of nonlinear models were determined by a trial-and-error method of computational operations. By minimizing the squared deviation and the coefficient of determination between the observed experimental data and the expected values during computer simulations, the parameters were determined.

### 5.6. In Silico Studies of AFB1 onto the Binding Surfaces of Chlorophyll-Amended Clay

We used MD simulations to investigate the binding of aflatoxin B1 (AFB1) to chlorophyll-amended clay (CMCH), as well as to parent (unamended) clay (CM) at acidic and neutral conditions. The AFB1 model structure was taken from PubChem (ID: 186907), chlorophyll was taken from PDB (molecule entry CLA) [43,44], while two layers of montmorillonite clay in acidic conditions were constructed, in accordance with our previous study [24], using CHARMM-GUI [45,46,47,48]. A separation distance of 21 Å was used between the clay layers, while the Na^+^ were removed and Ca^2+^ were introduced in random positions within the box. The edges of the clay layers were modified as in our previous studies [22,24] to simulate the clay model in neutral conditions. Topology and parameters for clay were taken from INTERFACE FF [49] in conjunction with CHARMM-GUI [45,46,47,48] for AFB1 from CGenFF [50], while for chlorophyll, a combination of CGenFF [50] and parameters from Guerra et al. [51] were used, as in our previous studies [22,24]. The input files for the preparation and simulation setup, used to (a) amend the clay with chlorophyll, and subsequently (b) test the binding of AFB1 to CMCH and CM, were initially generated by CHARMM-GUI [45,46,47,48], and were partially modified by us. The preparation of systems in both (a) and (b) was performed using CHARMM [46] and a 100 Å^3^ cubic water box. In (a), acidic clay conditions were used, with CM being centered and 12 copies of chlorophyll randomly being placed. The final simulation structure from (a) was used as an initial structure to prepare setup of (b), at which 12 copies of AFB1 randomly being placed away from, and without clashing with, clay or chlorophyll molecules. In (b), triplicate runs were performed, with clay represented independently at both acidic and neutral conditions. The simulations for both (a) and (b) were performed using OpenMM [52]. An equilibration (NVT) was performed at 300 K for 200 ps before the production (NPT) at 300 K and 1 atm (isotropic barostat) for 100 ns for (a) and 200 ns for (b), and in all cases magnesium/aluminum atoms of clay were constrained, analogously to our previous studies [22,24]. Upon completion of the simulations described in (b), analysis was performed with in-house programs to calculate the binding percentage probabilities of AFB1 to CM and CMCH and to understand and provide insight into the mechanisms of binding. This was conducted by decomposing chlorophyll into two chemical groups, the chlorin ring (referred to as “head”) and the alkyl chain (referred to as “tail”). The binding percentage probability was calculated by counting the total number of instances that an AFB1 molecule was in contact with the parent and amended clays, and it was normalized by the total number of AFB1 molecules in the simulations and the total number of snapshots analyzed. Subsequently, the average binding percentage probability was calculated from the triplicate runs for each system. Two entities were considered to be in contact, and thus interact with each other, when the distance between any pair of their atoms (including H) was ≤3.5 Å. Also, interactions between AFB1 and CM or CMCH were decomposed into direct, direct-assisted, and indirect-assisted interactions in accordance with our studies [22,24].

### 5.7. Cell Culture and Treatment

Human hepatocellular carcinoma (Hep G2) cells were cultured in Dulbecco’s Modified Eagle Medium (DMEM) supplemented with 10% fetal bovine serum (FBS), 1% non-essential amino acids, and 1% antibiotic-antimycotic. Cells were maintained on a collagen-coated surface (PureCol^®^ collagen at a concentration of 50 µg/mL) at 37 °C in a 5% CO_2_ atmosphere and subcultured once they reached 70–80% confluency. For the experiments, cells were seeded on collagen-coated 12-well plates at a density of 2 × 10^5^ cells per well and allowed to adhere overnight, or for 12 to 24 h. The cells were then exposed to various treatments consisting of vehicle control, aflatoxin B1 suspended in media, and clay-treated media containing aflatoxin B1. The vehicle control consisted of basal DMEM. Aflatoxin B1 was dissolved in acetonitrile and added to basal media to achieve a final concentration of 20 ppm. An aliquot of the aflatoxin B1 suspension was reserved to serve as the aflatoxin B1 treatment condition. For clay treatment, parent clays (CM and SM) and the GECs (CMCH, CMHCIN, SMCHIN, and SMCH) were tested each at a 0.2% (*w*/*v*) and 0.5% (*w*/*v*) inclusion rate in 2 mL of 20 ppm AFB1. The mixture was shaken on an electric shaker for two hours at 37 °C and 1000 rpm after which they were centrifuged for 20 min at 2000× *g*. The aliquots of the supernatants were reserved to serve as clay-treated exposure conditions. All samples were sterile filtered using a 0.22 µm syringe filter to reduce the possibility of contamination. Complete media were aspirated from each well of the seeded 12-well plates and 1 mL of each exposure condition was overlayed onto the cells and allowed to incubate for 24 h. After a 24 h exposure period, the spent media were collected and stored at −80 °C until further analysis. The exposure experiment was repeated three times on three separate occasions for a total of three biological replicates per treatment group. Each biological replicate consisted of separate cultures of cells treated with the respective conditions.

#### 5.7.1. Cytotoxicity Assessment

Cytotoxicity was assessed using the Lactate Dehydrogenase (LDH) Assay Kit (Colorimetric) (Cat. No. ab102526, Abcam, Inc., Cambridge, MA, USA) according to protocol Version 15a using the spent media from each exposure experiment.

#### 5.7.2. Statistical Analysis

Data were analyzed using GraphPad Prism (Version 10.3.0). Statistical significance between treatments was assessed by one-way ANOVA comparing the mean of each condition with the mean of the vehicle control followed by Dunnett’s post hoc test for multiple comparisons. A *p*-value of <0.05 was considered statistically significant. Data are presented in Figure 6 as mean ± standard deviation (SD) of the three biological replicates.

### 5.8. Hydra Vulgaris In Vivo Assay

Hydra vulgaris is a recognized in vivo model for examining the toxicity of mycotoxins [23,24,53,54]. Environmental Canada in Montreal supplied the Hydra vulgaris organisms used in this study, which were bred at a consistent temperature of 18 °C in hydra medium that contained 4 mg/L EDTA, 115 mg/L N-tris[hydroxymethyl]methyl-2-aminoethanesulfonic acid (TES), and 147 mg/L CaCl_2_ in 18.2 MΩ water that was adjusted to pH 6.9–7.0. Using an established hydra classification scoring system ranging from 0 to 10, hydra morphology serves as a biomarker for estimating the toxicity of chemicals under a dissecting microscope. This system assigns a score of 0 to a hydra that are thought to be dead or dissolved, and a score of 10 to those that are healthy and have long tentacles. Hydra were treated at different AFB1 concentrations ranging from 10 to 320 ppm for 92 h in order to investigate the toxicity profile of AFB1. The detoxification effects of the GECs were evaluated by treating 20 ppm of AFB1 with inclusions from 0.05% (*w*/*v*) to 0.5% (*w*/*v*) of the parent and the amended clays in hydra media following exposure to the hydra colony for 92 h. Two hydra colonies each in 2 mL of testing media at 18 °C made up each of the experimental groups.

Each experiment conducted in this study, including the negative and blank controls, was carried out at least three times. A post hoc Tukey test was used after a one-way ANOVA was used to determine the data’s statistical significance. Scores from toxicity and detoxification evaluations of hydra, as well as important metrics like Kd and Qmax obtained from adsorption isotherms, were examined for standard deviation and *p*-values. The results were deemed significant at *p* < 0.05.

## Figures and Tables

**Table 1:** Parameters and correlation coefficients of adsorption isotherms of AFB1 on binding surfaces of the clays.

Sorbents	Adsorption Parameters @ pH2				Adsorption Parameters @ pH6			
	Q_max_	K_d_	∆G	r^2^	Q_max_	K_d_	∆G	r^2^
CM	0.34	1.89 × 10^4^	−19.19	0.88	0.35	2.07 × 10^4^	−19.83	0.93
CMCH	0.43	2.01 × 10^4^	−20.28	0.95	0.41	1.86 × 10^4^	−19.95	0.96
CMCHin	0.40	2.18 × 10^4^	−20.07	0.96	0.36	2.09 × 10^4^	−19.71	0.96
SM	0.32	2.40 × 10^4^	−19.72	0.92	0.31	1.91 × 10^4^	−19.32	0.93
SMCH	0.38	1.66 × 10^4^	−19.60	0.97	0.33	2.68 × 10^4^	−19.71	0.94
SMCHin	0.39	1.14 × 10^4^	−19.33	0.94	0.38	1.63 × 10^4^	−19.83	0.92

Q_max_: binding capacity (mol/kg); K_d_: binding affinity; ∆G: Gibbs free energy (kJ/mol); r^2^: correlation coefficients. SMCHin; chlorophyllin-amended sodium montmorillonite; SMCH: chlorophyll-amended sodium montmorillonite; SM: sodium montmorillonite; CMCHin; chlorophyllin-amended calcium montmorillonite; CMCH: chlorophyll-amended calcium montmorillonite; CM: calcium montmorillonite.

**Table 2:** Adsorption kinetic parameters for AFB1 on the clays.

Pseudo-Second Order Parameters	qe (exp mgkg^−1^)	qe (cal mgkg^−1^)	K_2_	r^2^
CM	0.05	0.04	8.32 × 10^0^	0.85
CMCH	0.08	0.05	3.41 × 10^0^	0.87
CMCHin	0.04	0.04	1.36 × 10^0^	0.93
SM	0.05	0.04	3.66 × 10^0^	0.89
SMCH	0.07	0.05	1.36 × 10^0^	0.94
SMCHin	0.07	0.06	2.75 × 10^−1^	0.84

qe (exp): binding at equilibrium in experiment; qe (cal): binding at equilibrium calculated by pseudo-second order; K_2_: rate constant of the second order; r^2^: correlation coefficients.

## Data Availability

The raw data supporting the conclusions of this article will be made available by the authors on request.
